# The Vulnerability of the Older Child: A New Approach to Identifying Ages When Children Are Most Susceptible to Lead Effects

**DOI:** 10.1289/ehp.117-a358a

**Published:** 2009-08

**Affiliations:** **Tina Adler** first wrote for *EHP* about the Clinton–Gore environmental agenda in 1993. She is a member of the National Association of Science Writers and the American Society of Journalists and Authors

Watch a toddler, and you quickly see why small children are so vulnerable to environmental exposures: most of what they touch goes right into their mouths. It’s no wonder, then, that lead levels generally peak at age 2 years, which is when health officials recommend children be tested for elevated blood lead. But new research shows that 5- to 6-year-olds may be particularly vulnerable to the cognitive and behavioral effects of lead and should be tested as well if such problems are apparent **[*EHP*** **117:1309–1312; Hornung et al.]**.

Previous studies have suggested that IQ scores at ages 5–7 years are more strongly associated with concurrent blood lead concentrations than with concentrations measured at age 2. However, children’s blood lead concentrations during infancy are strongly associated with concentrations at older ages—meaning, for instance, a highly exposed toddler still tends to be highly exposed at age 6. This “serial correlation” makes it difficult to determine whether lead has a cumulative effect or whether effects of lead differ according to age.

In the current study, researchers analyzed blood lead concentration data for 462 children who participated in either the Cincinnati Lead Study, which enrolled children from 1979 to 1984, or the Rochester Longitudinal Study, which enrolled children from 1994 to 1995. In both studies the children’s blood lead was measured every year from infancy to age 6. The children also took IQ tests around age 6.

To study effects of lead at different ages while accounting for correlations in lead levels over time, the researchers estimated effects of the ratio of the child’s blood lead at age 2 relative to his or her blood lead at each subsequent age (3–6 years). As the ratio of age 6:age 2 blood lead increased, IQs declined even after controlling for average lead exposure at all ages as well as a range of other covariates. In addition, children who had relatively higher lead exposure at age 5 or 6 compared with age 2 had significantly higher arrest rates for criminal behavior in adulthood than other children.

The results suggest that blood lead testing and efforts to reduce exposure should continue as children reach school age. Moreover, lead testing of school-age children with cognitive or behavioral problems may help identify underlying causes of difficulties teachers or parents are seeing.

## Figures and Tables

**Figure:**
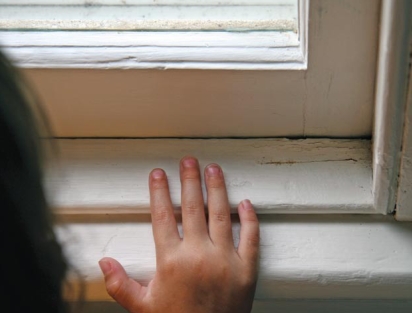
Although blood lead concentrations tend to decline after toddlerhood, older children may be more susceptible to adverse effects of exposure.

